# Peripheral artery disease In West Africans with diabetes: a risk factor profile analysis

**DOI:** 10.1016/j.ijcrp.2025.200469

**Published:** 2025-07-11

**Authors:** Joachim Amoako, Matthan Fayia Saa, Emmanuel Bannerman-Williams, Anastasia Naa Koshie Bruce, Maame Boatemaa Ansong, Alexander Danquah, Abraham Ablorh, Wills Nii Adjetey Kwaw, Michael Adjei, Emmanuel K. Awuttey, Isabella D. Dakubo, Patience Akos Vormatu, Isaac Ekow Ennin, Charles Frederick Hayfron-Benjamin

**Affiliations:** a Department of Surgery, University of Ghana Medical School, Ghana^1^; b Department of Physiology, University of Ghana Medical School, Ghana^1^; c College of Health Sciences University of Liberia, Liberia^1^; d Departments of Vascular Medicine, Amsterdam UMC, University of Amsterdam, Cardiovascular Sciences, Amsterdam, the Netherlands^1^; e Department of Public Health, Amsterdam UMC, University of Amsterdam, Amsterdam Public Health Research Institute, Amsterdam, the Netherlands^1^; f Department of Anesthesia and Critical Care, Korle Bu Teaching Hospital and University of Ghana Medical School, Ghana^1^; g School of Medical Sciences, University of Cape Coast, Ghana^1^; h Department of Pediatrics, Korle Bu Teaching Hospital, Ghana^1^; i Department of Physician Assistantship Studies, School of Medical Sciences, Central University, Ghana^1^; j Department of Anesthesia and Intensive Care, University of Ghana Medical Centre, Ghana^1^

**Keywords:** Diabetes, Peripheral artery disease, Risk factors, West Africans, Atherosclerosis

## Abstract

**Background:**

Globally, peripheral artery disease (PAD) affects >200 million people, disproportionately affecting people with diabetes. Prior studies characterizing the risk profile of PAD in diabetes have excluded West Africans, whose vascular biology is relevantly different. This study characterized the aggregate effect of modifiable risk factors on PAD in West Africans with diabetes.

**Methods:**

This was a cross-sectional study among 803 Ghanaian adults with diabetes. PAD was defined as ankle-brachial pressure index ≤0.90 and/or intermittent claudication. A multivariate logistic regression model was built to identify modifiable PAD risk factors, which were used to define the number of risk factors for each participant. The odds of PAD were determined based on the number of modifiable risk factors.

**Results:**

The mean age, diabetes duration, and HbA_1_c concentrations were 59.81(±9.95) years, 13.66(±7.89) years, and 8.45(±1.94) %, respectively. PAD prevalence was 25.8 %. In a multivariable regression model, hypertension [odds ratio 2.00, 95 % confidence interval 1.33–3.01], chronic kidney disease [1.54(1.11–2.14)], central obesity [1.58(1.05–2.39)], and elevated LDL-cholesterol concentration [1.42(1.02–1.97)] were independently associated with PAD. After adjustment for age, sex, and diabetes duration, the odds of PAD increased with each additional risk factor from a 2.02-fold increase [OR 2.02, 95 %CI 0.69–5.97) in the presence of two risk factors, to 3.51-fold [3.51(1.20–10.24)] for three risk factors, and nearly five-fold [4.80 (1.57–14.67)] for four risk factors.

**Conclusion:**

West Africans with diabetes are very sensitive to the cumulative effect of hypertension, chronic kidney disease, central obesity, and elevated LDL cholesterol concentration for PAD. These findings provide data to guide PAD screening/treatment strategies.

## Abbreviations

BMI: body mass index
CI: confidence interval
HbA_1_c: glycated hemoglobin
HDL: high-density lipoprotein
LDL: low-density lipoprotein
OR: odds ratio
PAD: peripheral artery disease

## 1 Introduction

Peripheral artery disease (PAD) is a progressive disease often characterized by obstructive atherosclerosis in the peripheral vasculature, typically arteries of the lower extremities [1]. Globally, PAD is highly prevalent, affecting over 200 million people [2] and about 10 % of adults aged ≥55 years [3]. In advanced stages, PAD may further complicate intermittent claudication, ischemic rest pain, ischemic ulcers/gangrene, and limb amputations [4]. These PAD-related complications are associated with repeated hospitalizations, increased healthcare-related costs, and early mortality [5]. Diabetes mellitus is a robust risk factor for PAD, being second only to cigarette smoking in contributing to PAD [6].

Existing reports show important ethnic differences in the burden and associated factors for PAD [7]. The PAD rate in African Americans is about twice that of non-Hispanic Whites at any given age [2]. African Americans are also known to be highly susceptible to the effects of conventional cardiovascular risk factors, compared with non-Hispanic Whites [8]. For example, in the presence of one conventional modifiable risk factor for PAD, the odds of PAD are over three times higher in African Americans than in non-Hispanic Whites [8]. The relationship between the presence of cardiovascular risk factors and the magnitude of PAD risk is complex due to the potential role of ethnicity. In Hispanic Americans, there is a higher prevalence of conventional cardiovascular risk factors compared with non-Hispanic Whites, but they do not have higher rates of PAD [9].

While over two-thirds of individuals with PAD live in low to middle-income countries [6], studies characterizing the burden and associated factors, as well as clinical profiles, including cumulative risk factor profiles for PAD in many of these populations, are extremely limited [1]. In West Africans with diabetes, studies assessing the predictive roles of modifiable risk factors for PAD, as well as their cumulative effects, are unavailable. Extrapolating from high-income settings using data from individuals of African ancestry to Africans living in Africa may be problematic, given the complex evolutionary history of Africans and African Americans [10] as well as the impact of environmental factors on cardiovascular disease [1]. Further, smoking, which is a major modifiable risk factor for PAD, has a low prevalence in many sub-Saharan African populations, including Ghana [11]. Given these, PAD risk factor studies in these often-neglected populations are imperative. The primary aim of this study is to characterize the aggregate effect of modifiable cardiovascular risk factors on PAD in a population of West Africans with diabetes. For this, we used a representative sample of Ghanaian individuals from various Ghanaian ethnic groups (Including Akan, Ewe, Ga/Dangbe, Mole/Dagomba, Mamprusi, Gruma, and Guan ethnic groups) managed at the National Diabetes Management and Research Centre in Accra Ghana. These ethnic groupings have shared ancestries and cultural practices with other West Africans, especially Ghana's neighboring countries.

## 2 Methods

### 2.1 Study design

Data for the current analyses are from two identical studies conducted at the University of Ghana Medical School/Korle Bu Teaching Hospital. The studies are identical for the methods (study population, questionnaires/surveys used, procedures/protocols for measurements, and equipment/devices used for measurement of ankle brachial pressure index, intermittent claudication, and covariates). The two studies differ only in the period of data collection. The first study took place from 2019 to 2022 and has been previously described [12]. The second study was from 2022 to 2024. Both studies included Ghanaians with established diagnoses of diabetes with no primary heart or lung disease or previous/current heart failure. Participants were excluded if they were pregnant. In the current analyses, participants were excluded if they were ineligible for the conduct of the ankle-brachial pressure index (ABI) measurements due to factors such as lower limb amputations. The flow chart (Supplementary Fig. 1) details inclusion in the current analyses. The current analyses include 803 unique participants with data on ankle-brachial pressure index (ABI) measurements and intermittent claudication based on the WHO Rose Angina Questionnaire [13]. Ethical approval of the study was obtained from the Ethics Committees/Institutional Review Boards of the University of Ghana College of Health Sciences (CHS-Et/M6-P2.14/2017–2018) and the Korle Bu Teaching Hospital (KBTH-IRB/000124/2019 for the first study/KBTH-STC/IRB/00037/2021 for the second study). All participants provided written informed consent before enrolment into the study.

### 2.2 Baseline measurements and covariates

The assessment of baseline characteristics, including sociodemographic characteristics, anthropometry, hemodynamic measurement, and biochemical measurements (including the concentrations of fasting plasma glucose, lipids, and HbA_1_c) has been previously described [14,15]. The surveys used in assessing the baseline characteristics are based on the World Health Organization (WHO) STEPwise approach to noncommunicable disease (NCD) surveillance (STEPS) which is validated in many populations, including Ghanaians, for NCD risk factor surveillance [16]. Diabetes duration was retrieved from the medical records and confirmed by the study survey.

Based on existing literature [6,8,17,18], six conventional modifiable cardiovascular risk factors for PAD were included in the current analyses, namely cigarette smoking, obesity/central obesity, hypertension, dyslipidemia, suboptimal glycemic control, and chronic kidney disease. Smoking was self-reported and was classified as never smokers, current smokers, and previous smokers. Due to the low number of ever smokers (current and previous smokers - 40 out of 803 study participants), current and previous smokers were combined into one category. Smoking pack years were used to quantify smoking. The standing heights (without shoes) were measured using the Seca 217 stadiometer and recorded to the nearest 0.1 cm. Weights were measured with study participants barefoot and in light clothing using the SECA 877 scale and recorded to the nearest 0.1 kg. Body mass index (BMI) in kg/m^2^ was calculated as the participant's body weight (in kg) divided by the square of his or her height (in m). Based on BMI, obesity was defined as BMI ≥30 kg/m^2^ [19]. Waist circumference (WC) was measured with the SECA 201 girth circumference measuring tape at the midpoint between the lower margin of the lowest palpable rib (posteriorly) and the top of the iliac crest. Central obesity was defined as a WC ≥ 94 cm in men and ≥80 cm in women [20]. Hip circumference (HC) was measured with the Seca 201 girth circumference measuring tape at the widest point, measured at the level of the greater trochanters. Waist-to-hip ratio (WHR) was determined as the ratio of WC to HC and used to define central obesity. Central obesity based on WHR was defined as WHR >0.9 in men and >0.85 in women. Blood pressure (BP) was measured thrice using the Omron BP Monitor HEM-907XL device, with appropriate-sized cuffs, after at least 5 min of rest while seated. The mean of the last two BP measurements was used for the analyses. Hypertension was based on a clinical diagnosis code/documentation in the medical records, evidenced by documented systolic blood pressure ≥140 mmHg and/or diastolic blood pressure ≥90 mmHg, and/or being on antihypertensive medication treatment [21]. Suboptimal BP control was defined as systolic BP >/ = 130 mmHg and/or diastolic BP >/ = 80 mmHg [22]. Dyslipidemia was based on the presence of high total cholesterol (TC), high low-density lipoprotein (LDL) cholesterol, high triglycerides (TG) and or low HDL-C based on the European Guidelines on Cardiovascular Prevention (2012 guideline) as follows [23]: plasma TC ≥ 5.0 mmol/L, TG ≥ 1.7 mmol/L, LDL-C ≥3.0 mmol/L and/or HDL-C <1.0 mmol/L in men and <1.2 mmol/L in women. Chronic kidney disease was based on the presence of albuminuria and or estimated glomerular filtration rate (eGFR) < 60 ml/min/1.73 m^2^. Albuminuria was defined as urinary albumin to creatinine ratio ≥30 mg/g (category ≥ A2) according to the 2012 Kidney Disease: Improving Global Outcomes (KDIGO) guidelines [24]. The eGFR was calculated from the plasma creatinine levels using the race-neutral 2021 Chronic Kidney Disease Epidemiology Collaboration (CKD-EPI) equation [25]. Optimum glycemic control was defined as an HbA_1_c concentration of ≤7 %, based on the American Diabetes Association and European Association for the Study of Diabetes consensus algorithm; this cut-off value is associated with benefits, including reducing vascular complications [26,27].

### 2.3 Peripheral artery disease assessment

ABI measurements were performed in the supine position after at least 10 min of supine rest using a validated oscillometric device (Microlife WatchBP Office ABI, Switzerland) with appropriate-sized cuffs [28]. Systolic BP was measured twice in the right and left brachial arteries and twice in the right and left posterior tibial arteries. ABI on either side was calculated as the ratio of the lowest ipsilateral ankle systolic BP (numerator) to the highest arm systolic BP (denominator). The lowest of the left and right ABI measurements were used for analyses. ABI obtained by the oscillometric method using the Microlife WatchBP Office ABI obtained by the oscillometric method has been shown to correlate well with ABI acquired by Doppler ultrasound, with a 95 % agreement between the two methods in diagnosing PAD [29]. Intermittent claudication was assessed using the WHO Rose Angina Questionnaire [13]. The questionnaire was administered by a physician and trained biomedical scientists. Intermittent claudication was defined as pain that appeared in either leg in the calf, when the patient walked uphill or hurried, or when walking at an ordinary pace on level ground and meeting all the following criteria: (i) never beginning when standing still or sitting, (ii) did not disappear while walking and (iii) forced the participant to stop or slow down. PAD was defined as ABI ≤0.90 [30] and/or the presence of intermittent claudication [13].

### 2.4 Statistical analysis

Data were analyzed using IBM SPSS (Version 26) for Windows. Data with a normal distribution were presented as mean (± standard deviation whereas those not normally distributed were presented as median (interquartile range). Categorical data were presented as frequencies (percentages). Differences in characteristics between individuals with and without PAD were assessed by the chi‐square test with Yales's correction for continuity or Fisher's exact test for categorical variables, t‐test for continuous variables, or the Mann‐Whitney U‐test for variables not normally distributed.

Binary logistic regression was performed to assess the associations of potential predictor variable with PAD, with adjustment for age, sex, and duration of diabetes. To assess which of these factors were independently associated with PAD, a multivariate logistic regression model was built with a backward stepwise selection of the covariates, resulting in a subset of predictor variables included in the final model. The backward model selection approach allowed the removal of non-significant modifiable risk factors until all risk factors were significant (p ≤ 0.05). The model selection process was cross‐checked using a forward selection of the predictor variables. A modifiable cardiovascular risk factor of interest was considered to be a PAD determinant if it was independently associated with PAD at a p-value<0.05. We then used the significant modifiable cardiovascular risk factors to define the number of modifiable risk factors for each participant. The odds (OR) and 95 % confidence interval (95 % CI) for PAD were determined based on the number of modifiable risk factors, with adjustment for age, sex, and diabetes duration. All p-values presented are two-tailed. A statistical test of significance was set at a p-value<0.05.

## 3 Results

### 3.1 General characteristics

Table 1 summarizes the baseline characteristics of the study population. Compared with individuals without PAD, individuals with PAD were much older and had a higher mean diabetes duration, systolic blood pressure, WC, WHR, and LDL-cholesterol concentration. The proportions of individuals with hypertension and CKD were respectively 14 % and 13 % higher in individuals with PAD compared with individuals without PAD. While the proportions of individuals with obesity and central obesity based on WC are comparable in the two groups, the proportion of individuals with central obesity based on WHR was 9 % higher in individuals with PAD compared with individuals without PAD. The two groups were comparable with respect to sex distribution, highest education attained, and proportion of individuals on insulin or statin therapy. The proportion of current/previous smokers was low (5.0 %) and did not differ between the two groups. For the 40 study participants who previously or currently smoked, the pack-years of smoking in 38 of them ranged between 1.0 and 6.0; the remaining two had pack years of 13 and 20 (see Table 2).

**Table 1:** Baseline characteristics of participants with and without peripheral artery disease.

	All Participants	No Peripheral Artery Disease	Peripheral Artery Disease	p-value∗
N	803	596	207	
Age (years)	59.81 (±9.95)	58.86 (±10.05)	62.53 (±9.14)	<0.001
Sex (%)				0.447
Female	614 (76.5 %)	460 (77.2 %)	154 (74.4 %)	
Male	189 (23.5 %)	136 (22.8 %)	53 (25.6 %)	
Diabetes duration, years	13.66 (±7.89)	13.09 ((±7.50)	15.29 (±8.72)	0.001
Higher education (%)	448 (55.8 %)	327 (54.9 %)	121 (58.5 %)	0.417
Alcohol consumption (%)	232 (28.9 %)	162 (27.2 %)	70 (33.8 %)	0.076
Current/previous smoker (%)	40 (5.0 %)	28 (4.7 %)	12 (5.8 %)	0.578
Smoking pack years∗	0.000 (0.000)	0.000 (0.000)	0.000 (0.000)	0.659^#^
Systolic BP, mmHg	136.12 (±17.80)	135.09 (±17.48)	139.11 (±18.43)	0.005
Diastolic BP, mmHg	77.15 (±10.30)	77.48 (±10.13)	76.19 (±10.72)	0.120
Pulse rate, beats per minute	78.88 (±11.66)	78.69 (±11.49)	79.41 (±12.16)	0.450
Hypertension (%)	584 (72.7 %)	412 (69.1 %)	172 (83.1 %)	<0.001
Suboptimal blood pressure control	563 (70.1 %)	406 (68.1 %)	157 (75.8 %)	0.042
BMI, kg/m^2^	29.45 (±5.96)	29.44 (±6.15)	29.46 (±5.40)	0.975
Obesity (%)	319 (39.7 %)	227 (38.1 %)	92 (44.4 %)	0.117
Waist circumference, cm	98.88 (±13.38)	98.30 (±13.46)	100.53 (±13.04)	0.039
Central obesity based on WC (%)	691 (86.1 %)	510 (85.6 %)	181 (87.4 %)	0.581
Waist to hip ratio	0.92 (±0.10)	0.91 (±0.09)	0.94 (±0.12)	0.001
Central obesity based on WHR (%)	611 (76.1 %)	440 (73.8 %)	171 (82.6 %)	0.011
eGFR, ml per minute	82.49 (±25.29)	84.82 (±25.05)	76.14 (±24.92)	<0.001
Urinary ACR, mg/g∗	21.00 (41.50)	19.30 (40.70)	24.00 (57.00)	0.112^#^
Chronic Kidney Disease (%)	303 (37.7 %)	205 (34.4 %)	98 (47.3 %)	0.001
HbA_1_c, %	8.45 (±1.94)	8.39 (±1.95)	8.64 (±1.92)	0.107
HbA_1_c > 7 %	618 (77.0 %)	451 (75.7 %)	167 (80.7 %)	0.151
Insulin therapy (%)	326 (40.6 %)	235 (39.4 %)	91 (44.0 %)	0.286
Statin therapy (%)	385 (47.9 %)	293 (49.2 %)	92 (44.4 %)	0.259
Total cholesterol, mmol/l	4.96 (±1.26)	4.90 (±1.16)	5.12 (±1.49)	0.055
Triglyceride, mmol/l	1.08 (±0.52)	1.10 (±0.52)	1.03 (±0.53)	0.078
HDL- cholesterol, mmol/l	1.42 (±0.44)	1.41 (±0.43)	1.45 (±0.47)	0.393
LDL-cholesterol, mmol/l	3.04 (±1.15)	2.98 (±1.09)	3.20 (±1.29)	0.028

Values for categorical variables are given as number (percentage); for continuous variables, as mean (±standard deviation) or median (interquartile range).

∗T test for mean difference or Fisher's exact test for percentage difference, unless otherwise indicated.

#Mann-Whitney *U* test.

eGFR in ml/min is standardized for body surface area. eGFR is based on the race-neutral 2021 CKD-EPI eGFR equations.

Definitions of variables.

Central obesity (based on WC) was defined as waist circumference ≥94 cm in men and ≥80 cm in women.

Central obesity (based on WHR) was defined as WHR >0.9 in men and >0.85 in women.

Chronic kidney disease is based on the presence of albuminuria (urinary albumin to creatinine ratio ≥30 mg/g) and/or eGFR <60 ml/min/1.73 m^2^.

Current/previous smoking was self-reported.

Elevated LDL cholesterol concentration was based on values ≥ 3.0 mmol/L.

Hypertension was based on a clinical diagnosis code, systolic blood pressure ≥140 mmHg and/or diastolic blood pressure ≥90 mmHg, and/or being on antihypertensive medication treatment.

Higher education is defined as secondary education and above.

Definition of abbreviations.

BMI = Body mass index; BP = blood pressure; HbA_1_c = glycosylated hemoglobin; HDL = high-density lipoprotein; LDL = low-density lipoprotein.

∗ Values expressed in median (interquartile range).

**Table 2:** Multivariate logistic regression models for traditional cardiovascular risk factors associated with peripheral artery disease.

	Odds Ratio	95 % Confidence Interval	p-value
Hypertension	2.00	1.33–3.01	0.001
Chronic kidney disease	1.54	1.11–2.14	0.009
Central obesity based on WHR	1.58	1.05–2.39	0.028
Elevated LDL cholesterol concentration	1.42	1.02–1.97	0.036

Definitions of variables.

Central obesity (based on WC) was defined as waist circumference ≥94 cm in men and ≥80 cm in women.

Central obesity (based on WHR) was defined as WHR >0.9 in men and >0.85 in women.

Chronic kidney disease is based on the presence of albuminuria (urinary albumin to creatinine ratio ≥30 mg/g) and/or eGFR <60 ml/min/1.73 m^2^.

Current/previous smoking was self-reported.

Elevated LDL cholesterol concentration was based on values ≥ 3.0 mmol/L.

Hypertension was based on a clinical diagnosis code, systolic blood pressure ≥140 mmHg and/or diastolic blood pressure ≥90 mmHg, and/or being on antihypertensive medication treatment.

Definition of abbreviations.

HbA_1_c = glycosylated hemoglobin; LDL = low-density lipoprotein; WHR = waist to hip ratio.

### 3.2 PAD prevalence

**Fig. 1:**
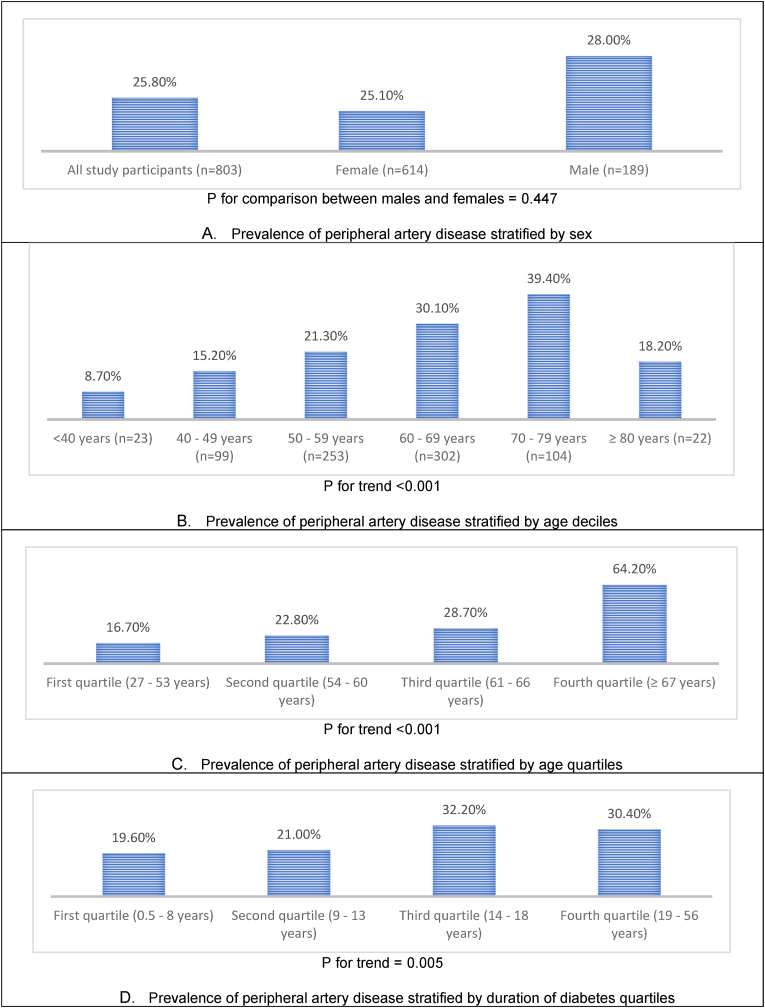
Prevalence of peripheral artery disease stratified by major non-modifiable PAD risk factor groupings in represents the number of participants in each group.

Fig. 1 shows the prevalence of PAD stratified by sex, age, and diabetes duration. In the overall study population, the prevalence of PAD was 25.5 %. This did not significantly differ by sex (28.0 % in males and 25.1 % in females, p = 0.447). The prevalence of PAD increased across age deciles and quartiles. Across age deciles up to 79 years, the prevalence of PAD increased by at least 6 percent per decile. In our study population, individuals aged 70–79 years had the highest prevalence of PAD, with four out of 10 persons affected. Individuals aged 60 years (mean age of the study population) and above had a higher prevalence of PAD compared with those aged below 60 years (29.0 % vs. 17.9 %, p < 0.001). The PAD prevalence varied across the diabetes duration quartile, increasing from the first to the third quartile. Individuals with diabetes 14 years (mean diabetes duration of the study population) and above had a higher prevalence of PAD compared with those with diabetes duration below 14 years (31.3 % vs. 20.3 %, p < 0.001).

### 3.3 Modifiable risk factors for PAD

Supplementary Table 1 shows the associations between the potential modifiable PAD risk and PAD. In the crude model, hypertension, CKD, central obesity based on WHR, and elevated LDL cholesterol concentrations were associated with higher odds of CKD. Cigarette smoking, suboptimal glycemic control, and other measures of obesity/central obesity and dyslipidemia were not significantly associated with higher odds of PAD. Similar observations were made in models adjusted for age and sex. In models adjusted for age, sex, and diabetes duration, hypertension [odds ratio 1.83, 95 % CI 1.21–2.78, p-value = 0.004], CKD [1.51 (1.08–2.10), 0.015], central obesity based on WHR [1.74 (1.15–2.64), 0.009], obesity based on BMI [1.44 (1.02–2.03), 0.036], and elevated LDL cholesterol concentrations [1.45 (1.05–2.01), 0.026] were associated with higher odds of PAD. In a multivariable regression model, hypertension [2.00 (1.33–3.01), 0.001], CKD [1.54 (1.11–2.14), 0.009], Central obesity based on WHR [1.58 (1.05–2.39), 0.028], and elevated LDL cholesterol concentration [1.42 (1.02–1.97), 0.036] were independently associated with higher odds of PAD.

### 3.4 Cumulative risk factor profiles and odds of PAD

Supplementary Table 2 shows the distribution of PAD prevalence by the cumulative number of modifiable PAD risk factors. PAD was present in 11.8 % of the study population with diabetes without any additional modifiable PAD risk factors. Individually or together, hypertension, CKD, central obesity (based on WHR), and/or elevated LDL cholesterol concentration explained 88.2 % of all cases of PAD. Respectively, 12.5 %, 21.2 %, 31.8 %, and 39.0 % of the participants had one, two, three, or all four independent modifiable PAD risk factors. An increasing number of PAD risk factors were associated with higher odds of PAD (p < 0.001). Supplementary Table 3 shows the distribution of PAD prevalence by specific combinations of modifiable PAD risk factors.

**Fig. 2:**
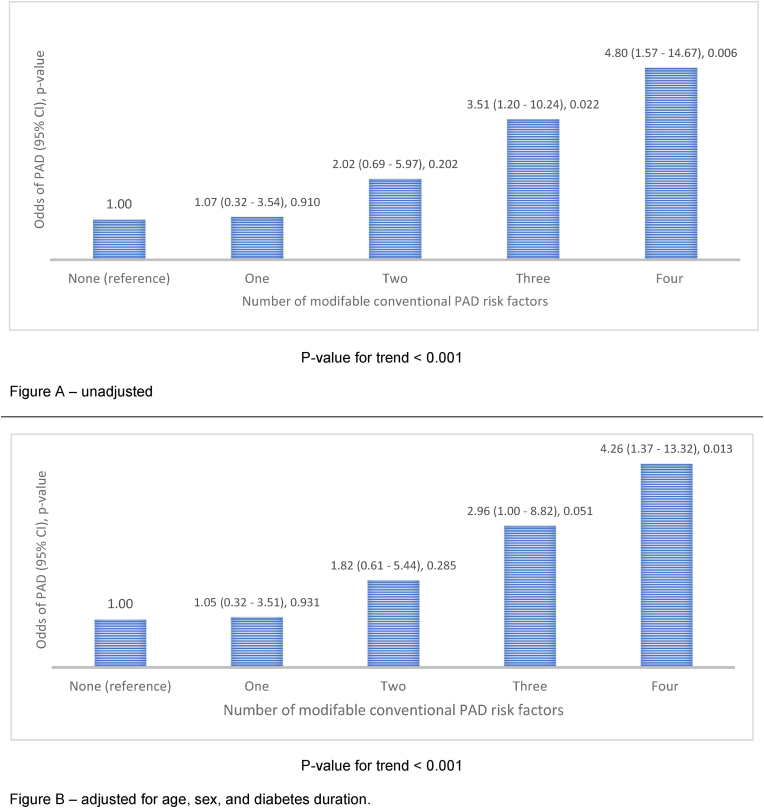
Relative odds of peripheral arterial disease (PAD) based on the number of modifiable PAD risk factors, The reference group is individuals without any of the modifiable conventional PAD risk factors (hypertension, chronic kidney disease, central obesity, and elevated LDL cholesterol concentration), Odds ratios (95 % confidence intervals) and trend probabilities of PAD based on an increased number of modifiable PAD risk factors (hypertension, chronic kidney disease, and elevated LDL cholesterol concentration).

In analyses that explored the cumulative impact of modifiable cardiovascular risk factors on PAD, an increasing number of modifiable risk factors was associated with higher odds of PAD (p < 0.001 for the trend) for both unadjusted and fully adjusted models (Fig. 2A and B). In the unadjusted model, one risk factor present relative to no risk factors did not increase the odds of PAD (odds ratio 1.07, 95 % CI 0.32–3.54, p-value = 0.910). With two risk factors present relative to no risk factors, the risk for PAD was over twice as high, although this was not statistically significant 2.02 (0.69–5.97), 0.202]. For three risk factors relative to no risk factor, the risk for PAD was over thrice as high [3.51 (1.20–10.24), 0.022], and for four risk factors, the risk was increased nearly fivefold [4.80 (1.57–14.67), 0.006]. In a model adjusted for sex, age and duration of diabetes, the presence of three risk factors tended to increase PAD risk by nearly three-fold [2.96 (1.00–8.82), 0.051],while the presence of four risk factors increased PAD risk by over four-fold [4.26 (1.37–13.32), 0.013]

## 4 Discussion

### 4.1 Summary of key findings

In this study population of West Africans with diabetes with a low smoking index, PAD was prevalent, affecting 25.8 % of the study population. PAD prevalence did not differ by sex but generally increased with increasing age or duration of diabetes. Four conventional modifiable risk factors (hypertension, chronic kidney disease, central obesity based on elevated WHR, and elevated LDL cholesterol concentrations) were independently associated with PAD, with hypertension and chronic kidney disease being the most robust in a model adjusted for age, sex, and diabetes duration. Individually or together, these four risk factors explained 88.2 % of all cases of PAD. A simplified score based on these four conventional cardiovascular risk factors showed that West Africans with diabetes are sensitive to the cumulative effect of PAD risk factors.

### 4.2 Discussion of key findings

The current study is not the first to report the prevalence of PAD in a general sub-Saharan African population or among sub-Saharan Africans with diabetes [[31], [32], [33]]. In the meta-analysis by Johnston et al., PAD prevalence was 39 %–52 % in sub-Saharan Africans with known risk factors like diabetes [31]. The lower prevalence of PAD in the current study compared with the prevalence reported in the meta-analysis by Johnston et al. could reflect enhanced diabetes and cardiovascular care obtained by this cohort managed at a National Diabetes Management and Research Centre in Ghana. Our observed prevalence rates compare well with the typical PAD prevalence rate of 20 %–30 % in studies involving individuals with diabetes living in the United States and Europe [34].

In this study, the prevalence of PAD was similar in males and females. Data on sex differences in PAD in the setting of diabetes is limited. One study based on the Framingham study data showed that impaired glucose tolerance was a greater risk for PAD in women compared with men [35]. In studies not limited to individuals with diabetes, the reported relationship between sex and PAD risk is complex and tends to vary with factors including age and income/resource setting. In high-income settings, males tend to have higher PAD prevalence than females [6]. However, in low-income settings, PAD rates are known to be higher in females than in males [6]; this may be partly driven by environmental exposure differentially affecting women who live in low-income settings such as particulate matter, including particulate matter ≤10 μm in diameter (PM_10_), known to be associated with PAD [36]. Our finding that increasing age and duration of diabetes are associated with a higher likelihood of PAD is consistent with previous reports [3,37]. For age deciles, PAD prevalence consistently increased from <40 years to the 70–79 age group, after which it declined in the 80–90 year group. Similar observations were made for PAD prevalence stratified by diabetes duration quartiles, where PAD rates were higher in the third quartile than in the fourth quartile. These observations may reflect natural selection phenomena.

The current study shows that in West Africans with diabetes, hypertension, CKD, central obesity (based on WHR), and elevated LDL cholesterol concentration was independently associated with PAD and explained nearly nine in ten PAD cases. The results for hypertension, elevated LDL cholesterol concentrations, and central obesity agree with most prior studies in other ethnic groups [6]. Hypertension is a known established risk factor of both symptomatic and asymptomatic PAD based on large population-based studies [38,39]. Dyslipidemia is also an established risk factor for PAD, with elevated LDL cholesterol being the most robust indicator [40]. A notable contribution of this study to the existing literature is that among West Africans with diabetes, central obesity based on elevated WHR is better correlated with PAD than central obesity based on WC or peripheral obesity (obesity based on elevated BMI). Among individuals with diabetes, Rafsanjani and colleagues have reported that after adjusting for several confounding factors, including uric acid concentrations, pulse pressure, triglyceride concentrations, and eGFR, increased WHR was most strongly associated with atherosclerotic cardiovascular disease [41]. Why central obesity based on WHR correlates better with PAD than obesity based on BMI has a known biological basis. While peripheral obesity is characterized by increased subcutaneous fat, central obesity is characterized by increased visceral fat [42]. Visceral fat is more metabolically active than subcutaneous fat [42] and is thus more likely to drive atherosclerotic cardiovascular disease than subcutaneous fat does [43]. Specific mechanisms linking central obesity to PAD include deranged lipid metabolism and enhanced inflammation. For example, elevated concentrations of free fatty acids together with intra-abdominal fat accumulation, which occurs in central obesity, could drive insulin resistance, which drives atherosclerotic cardiovascular disease [41]. Visceral fat is more metabolically active than subcutaneous fat and likely to drive processes leading to the release of inflammatory biomarkers, including cytokines that may promote the recruitment of immune cells to the arterial wall to drive atherosclerosis [42]. However, why central obesity based on WHR better correlates with PAD compared with central obesity based on WC is uncertain and could relate to the accuracy of the ethnic-specific thresholds for normality recommended for sub-Saharan Africans [20]. Regarding assessment of central obesity, WHR has some advantages over WC, including its measurement errors being less associated with the size of the individual, as well as being less dependent on ethnicity [44]. Currently, there are no ethnic-specific values for WC to define central obesity in sub-Saharan Africans. The current guidelines recommend the use of European data until more specific data are available for sub-Saharan Africans [20]; this may introduce inaccuracies given the ethnic differences in body composition [20,45].

Our results show that CKD is a strong, independent modifiable risk factor for PAD in diabetes, second only to hypertension. Most prior studies assessing PAD risk factors have not specifically reported the role of CKD. However, studies not limited to individuals with diabetes have reported PAD as more prevalent in individuals with albuminuria or reduced eGFR [[46], [47], [48]]. The biological basis of the relationship between CKD and PAD is largely unexplored but could reflect shared risk factors (such as increasing age) or higher risk factors for PAD in patients with albuminuria and/or reduced eGFR, although prior studies have reported the persistence of this association after adjusting for these shared potential risk factors [49,50]. It could also be the case that toxins that build up in the extracellular fluid in the setting of CKD may adversely impact vascular biology. For example, uremic toxins may drive endothelial activation and increase the risk of atherosclerotic macrovascular complications, including PAD [51]. Together, these results on PAD modifiable risk factors suggest that appropriate lifestyle modifications and treatment targeting these modifiable risk factors could reduce the burden of PAD in West Africans with diabetes. Indeed, a previous meta-analysis showed improvements in leg ischemia in patients with PAD following antihypertensive therapy [52]. Similar results exist for interventions targeting dyslipidemia [53].

An unexpected finding in this study was that cigarette smoking was not associated with PAD. The relationship between smoking and PAD is robust [6], and has a mechanistic and epidemiological basis. Smoking is more strongly associated with PAD than any of the atherosclerotic cardiovascular diseases and is driven by mechanisms including enhanced inflammation, endothelial damage, arterial smooth muscle proliferation, and increased sympathetic tone [54,55]. A systematic review and meta-analysis that included 55 eligible studies performed in different ethnic groups demonstrated that both current and previous smoking are associated with PAD [56]. Why cigarette smoking was not associated with PAD in our study population is unclear, but could reflect the remarkably low cigarette smoking rate/index, which is representative of the national population smoking rate of 3.8 % [11]. When an established risk factor is rare, it becomes less likely to detect a statistically significant association with an outcome variable, even if a true causal relationship exists. Statistically, a limited number of individuals exposed to smoking reduces the statistical power to observe a difference in PAD occurrence between smokers and non-smokers. Besides, the relationship of measures of smoking with cardiovascular disease is dose-dependent, and the low pack-years of smoking in those who smoked could not have impacted their vascular biology. While the risk of PAD is not eliminated with low pack years of smoking, the damage and progression of atherosclerosis are likely to be less pronounced with low smoking pack years (which represents low cumulative exposure) [57]. Data from the study by Conen et al. showed that 10 pack-years of lifetime smoking exposure is required to increase the hazard ratio of PAD in individuals with no cardiovascular disease by 2.52. [58]. Further, some adverse effects of smoking on vascular biology are reversible with smoking cessation, and this may be especially true if the pack years of smoking is low [57].

To the best of our knowledge, no prior study has explored the cumulative effect of modifiable cardiovascular risk factors on PAD in sub-Saharan Africans, both in the general population and in individuals in specific disease groupings like diabetes. We report a simplified score based on four conventional modifiable cardiovascular risk factors that predict the likelihood of PAD among West Africans with diabetes. According to our results, an increasing number of risk factors generally increased PAD risk, but the association was more pronounced when at least two risk factors were present. Eraso et al. [8] had previously developed a similar PAD scoring system to evaluate the cumulative impact of cardiovascular risk factors on PAD risk in a population not limited to individuals with diabetes (using the United States National Health and Nutrition Examination Survey that included non-Hispanic Blacks, non-Hispanic Whites, and Mexican Americans). Similar to our study results, Eraso et al. observed an incremental trend in the odds of PAD that was proportional to the number of cardiovascular risk factors (hypertension, diabetes, hypercholesterolemia, current smoking, and chronic kidney disease). In the overall study population, the authors reported that for each additional risk factor present, the odds of PAD nearly doubled.

Broadly, our findings of the cumulative impact of risk factors on PAD suggest that West Africans with diabetes are sensitive to the cumulative effect of PAD risk factors, especially when the number of identified risk factors exceeds two. Our observation that one risk factor present relative to no risk factors did not increase the odds of PAD highlights the importance of diabetes itself as a major risk factor for PAD. Indeed, diabetes and smoking are known to be the strongest risk factors for PAD [37]. Based on our results, the impact of diabetes per se on PAD risk in West Africans may be equal to or exceed the risk posed by two of the modifiable risk factors in this study. For example, the PAD rate in individuals with diabetes with no additional risk factor was 11.8 %, while that in individuals with diabetes and two risk factors was 21.2 %. Future studies are required to confirm or refute this claim. If this claim is valid, then emphasis on diabetes prevention in this population with a low smoking index should be the principal strategy in driving down PAD risk. Our finding that West Africans with diabetes are particularly sensitive to the aggregate effect of PAD risk factors is of important clinical and public health significance in a low-resource setting. While all individuals with diabetes require periodic screening for vascular complications, including PAD, financial and logistical constraints [59] limit such screening in low-resource settings. This simplified score based on four easy-to-assess, modifiable, conventional cardiovascular risk factors may be valuable in prioritizing West Africans with diabetes for more frequent PAD assessment. In addition, the score may be valuable in PAD prevention and treatment programs.

While the minority of participants (11.8 %) with none of the identified modifiable risk factors had PAD, this proportion is clinically relevant and requires discussion. It is conceivable that aside from diabetes itself increasing PAD risk, other factors, aside from the conventional modifiable risk factors, could drive processes leading to PAD, including vascular inflammation, endothelial dysfunction, and thrombotic risk [34,60]. Of note, among them is the role of chronic or recurrent infections/infestations, which are commoner in people living in low to middle-income countries. Chronic infections/infestations may trigger inflammatory pathways, predisposing the arterial vessels of the peripheral vasculature to atherosclerosis [[61], [62], [63]]. Future studies could explore the impact of chronic infections/infestations on PAD in individuals with diabetes. Genetic factors can also not be excluded, given that PAD may result from an interaction of numerous genes and the environment.

### 4.3 Strengths and limitations

This study is novel because it provides quantitative evidence of the aggregate effect that cardiovascular risk factors have on PAD prevalence in West Africans with diabetes. The included risk factors in the simplified score are also easy to assess, even in low-income settings. Our study also reports on the differential impacts of individual risk factors on the likelihood of PAD. Our study has some limitations. First, the cross-sectional design limits making causal inferences. Secondly, smoking status and assessment of smoking pack years were self-reported which could have resulted in recall bias. However, this method of assessment is typically used in cardiovascular disease studies. Thirdly, we did not assess specific risk factor permutations when assessing the cumulative effect of the risk factors on PAD due to a limited number of participants in some of the permutation groupings. Fourthly, conventional arteriography, the gold standard for vascular imaging, and other advanced imaging modalities like computerized tomography and magnetic resonance angiography were not employed in the assessment of PAD due to feasibility. Albeit, ABI is known to correlate well with angiographically verified PAD [64]. Finally, the study was performed at a single study site, and included a relatively smaller number of study participants.

## 5 Conclusions

Among West Africans with diabetes, hypertension, CKD, central obesity based on WHR, and elevated LDL cholesterol concentration were independently associated with PAD. These modifiable cardiovascular risk factors were used to develop a simple PAD risk factor score that showed that each additional modifiable cardiovascular risk factor resulted in more than double the odds of PAD at each incremental level. These findings suggest that West Africans with diabetes are particularly sensitive to the cumulative effect of PAD risk factors and provide useful data to guide PAD screening and treatment strategies. Future larger studies could assess specific modifiable risk factor permutations with the greatest PAD risk.

## CRediT authorship contribution statement

**Joachim Amoako:** Writing – review & editing, Writing – original draft, Methodology. **Matthan Fayia Saa:** Writing – review & editing, Writing – original draft, Investigation. **Emmanuel Bannerman-Williams:** Writing – review & editing, Writing – original draft, Investigation, Funding acquisition, Data curation. **Anastasia Naa Koshie Bruce:** Writing – review & editing, Writing – original draft, Investigation, Formal analysis. **Maame Boatemaa Ansong:** Writing – review & editing, Writing – original draft, Investigation. **Alexander Danquah:** Writing – review & editing, Writing – original draft, Investigation. **Abraham Ablorh:** Writing – review & editing, Writing – original draft, Investigation. **Wills Nii Adjetey Kwaw:** Writing – review & editing, Writing – original draft, Investigation. **Michael Adjei:** Writing – review & editing, Writing – original draft, Investigation. **Emmanuel K. Awuttey:** Writing – review & editing, Writing – original draft, Investigation. **Isabella D. Dakubo:** Writing – review & editing, Writing – original draft, Methodology. **Patience Akos Vormatu:** Writing – review & editing, Writing – original draft, Project administration, Investigation, Data curation. **Isaac Ekow Ennin:** Writing – review & editing, Writing – original draft. **Charles Frederick Hayfron-Benjamin:** Writing – review & editing, Writing – original draft, Methodology, Investigation, Funding acquisition, Formal analysis, Data curation, Conceptualization.

## Funding

This work was funded by the Faculty Development Grant of the 10.13039/501100005601University of Ghana College of Health Science.

## Declarations of competing interest

None.
