# Supplementary material for: Peripheral artery disease In West Africans with diabetes: a risk factor profile analysis
Source: Int J Cardiol Cardiovasc Risk Prev. 2025 Jul 11;26:200469. doi: 10.1016/j.ijcrp.2025.200469 (PMC12281030; doi:10.1016/j.ijcrp.2025.200469)
Supplement: Multimedia component 1 [file mmc1.docx]

Figure 1: Flow chart of study design and inclusion in analysis.

Individuals approached to take part in the study (N=1211)

1049 agreed to take part in the study

162 did not agree to take part in the study

Excluded from the study due to primary heart or lung disease or previous/current heart failure (N=22)

Considered eligible for the umbrella studies the main studies that explores pulmonary and/or vascular function and their associated factors among Ghanaians with diabetes (N=1027)

Ineligible for evaluation of ABI (N=12)

Considered eligible for the current analyses aiming to characterized the aggregate effect of modifiable risk factors on PAD in West Africans with diabetes (N=1015)

Exclusion based on missing or incomplete data on

ABI and WHO RAQ (N=71)

Key covariates included in model

(N=141)

**Included in the final analyses (N=803)**

List of Abbreviations

ABI = Ankle Brachial Pressure Index

FPG = Fasting Plasma Glucose

HbA1c = Glycated Hemoglobin

RAQ = Rose Angina Questionnaire

WHO = World Health Organization
