# Supplementary material for: Peripheral artery disease In West Africans with diabetes: a risk factor profile analysis
Source: Int J Cardiol Cardiovasc Risk Prev. 2025 Jul 11;26:200469. doi: 10.1016/j.ijcrp.2025.200469 (PMC12281030; doi:10.1016/j.ijcrp.2025.200469)
Supplement: Multimedia component 2 [file mmc2.docx]

**Supplementary Table 1: Associations between the modifiable cardiovascular risk factors (independent variable) and peripheral artery disease (dependent variable)**

|  | Peripheral Artery Disease  Odds Ratio (95% Confidence Interval), p-value | | |
| --- | --- | --- | --- |
|  | Model 1 | Model 2 | Model 3 |
| Hypertension | 2.19 (1.47 -3.28), <0.001 | 1.87 (1.24 - 2.83), 0.003 | 1.83 (1.21 - 2.78), 0.004 |
| Measures of dyslipidemia |  |  |  |
| Elevated total cholesterol | 1.22 (0.89 - 1.69), 0.216 | 1.26 (0.91 - 1.75), 0.161 | 1.26 (0.91 - 1.75), 0.166 |
| Elevated triglyceride | 0.75 (0.44 - 1.30), 0.306 | 0.81 0.47 - 1.41), 0.455 | 0.82 (0.47 - 1.43), 0.486 |
| Low HDL cholesterol | 0.92 (0.64 - 1.33), 0.661 | 0.99 (0.68 - 1.44), 0.961 | 1.00 (0.69 - 1.45), 0.992 |
| Elevated LDL cholesterol | 1.40 (1.01 - 1.93), 0.041 | 1.42 (1.03 - 1.97), 0.034 | 1.45 (1.05 - 2.01), 0.026 |
| Current/previous smoking | 1.25 (0.62 - 2.50), 0.532 | 1.04 (0.49 - 2.18), 0.926 | 1.02 (0.49 - 2.16), 0.949 |
| Measures of obesity |  |  |  |
| Central Obesity based on WC | 1.17 (0.73 - 1.88), 0.504 | 1.36 (0.79 - 2.34), 0.264 | 1.37 (0.79 - 2.35), 0.259 |
| Central Obesity based on WHR | 1.68 (1.13 - 2.52), 0.011 | 1.76 (1.16 - 2.66), 0.008 | 1.74 (1.15 - 2.64), 0.009 |
| Obesity based on BMI | 1.30 (0.94 - 1.79), 0.108 | 1.44 (1.02 - 2.03), 0.036 | 1.44 (1.02- 2.03), 0.036 |
| Overweight/obesity | 1.17 (0.79 - 1.73), 0.422 | 1.28 (0.85 - 1.93), 0.231 | 1.28 (0.85 - 1.93), 0.230 |
| HbA_1_c > 7% | 1.34 (0.91 - 1.99), 0.142 | 1.48 (0.99 - 2.20), 0.057 | 1.44 (0.96 - 2.15), 0.079 |
| Chronic kidney disease | 1.71 (1.24 - 2.36), 0.001 | 1.53 (1.10 - 2.13), 0.011 | 1.51 (1.08 - 2.10), 0.015 |

Model 1 = unadjusted

Model 2 = adjusted for age and sex

Model 3 = Adjusted for age, sex, and diabetes duration.

Definitions of variables.

Central obesity (based on WC) was defined as waist circumference ≥94 cm in men and ≥80cm in women

Central obesity (based on WHR) was defined as WHR >0.9 in men and >0.85 in women

Chronic kidney disease is based on the presence of albuminuria (urinary albumin to creatinine ration ≥ 30mg/g) and/or eGFR < 60 ml/min/1.73 m^2^.

Elevated total cholesterol concentration ≥ 5.0 mmol/L; elevated triglyceride concentration ≥1.7 mmol/L; elevated LDL cholesterol concentration ≥3.0 mmol/L; low HDL cholesterol concentration <1.0 mmol/L in men and <1.2 mmol/L in women) .

Hypertension was based on a clinical diagnosis code, systolic blood pressure ≥140 mmHg and/or diastolic blood pressure ≥ 90 mmHg, and/or being on antihypertensive medication treatment.

Obesity was defined as a body mass index ≥ 30 kg/m^2^.

Overweight or obesity was defined as a body mass index ≥25 kg/m^2^

**Supplementary Table 2: Distribution of PAD prevalence by cumulative number of modifiable PAD risk factors**

|  | Entire Study Participants | No Risk Factor | One Risk Factor | Two Risk Factors | Three Risk Factors | Four Risk Factors |
| --- | --- | --- | --- | --- | --- | --- |
| No PAD | 596 (74.2%) | 30 (88.2%) | 91 (87.5%) | 215 (78.8%) | 199 (68.2%) | 61 (61.0%) |
| PAD | 207 (25.8%) | 4 (11.8%) | 13 (12.5%) | 58 (21.2%) | 93 (31.8%) | 39 (39.0%) |

P-value < 0.001

Risk factors included in the analyses are hypertension, elevated LDL cholesterol concentration, central obesity based on waist to hip ratio, and chronic kidney disease.

**Supplementary Table 3: Distribution of PAD prevalence by specific combinations of modifiable PAD risk factors**

| **Combination of PAD risk factors** | **No PAD** | **PAD** |
| --- | --- | --- |
| None | 30 (88.2%) | 4 (11.8%) |
| Hypertension only | 30 (81.1%) | 7 (18.9%) |
| CKD only | 10 (83.3%) | 2 (16.7%) |
| Central obesity only | 40 (90.9%) | 4 (9.1%) |
| Elevated LDL-C only | 18 (94.7%) | 1 (5.3%) |
| Hypertension and CKD only | 11 (84.6%) | 2 (15.4%) |
| Hypertension and central obesity only | 92 (76.0%) | 29 (24.0%) |
| Hypertension and elevated LDL-C only | 36 (83.7%) | 7 (16.3%) |
| CKD and elevated LDL-C only* | - | - |
| CKD and central obesity only | 18 (81.8%) | 4 (18.2%) |
| Central obesity and elevated LDL-C only | 51 (77.3%) | 15 (22.7%) |
| Hypertension, CKD, and central obesity only | 67 (67.0%) | 33 (33.0%) |
| Hypertension, CKD, and elevated LDL-C only | 21 (61.8%) | 13 (38.2%) |
| Hypertension, central obesity, and elevated LDL-C only | 94 (69.1%) | 42 (30.9%) |
| CKD, central obesity, and elevated LDL-C only | 17 (77.3%) | 5 (22.7%) |
| All four modifiable risk factors (Hypertension, CKD, central obesity and elevated LDL-C) | 61 (61.0%) | 39 (39.0%) |

* No study participant had CKD and elevated LDL-C only

*Definitions of variables.*

Central obesity was based on waist to hip ratio (WHR) and was defined as WHR >0.9 in men and >0.85 in women

Chronic kidney disease is based on the presence of albuminuria (urinary albumin to creatinine ration ≥ 30mg/g ) and/or eGFR < 60 ml/min/1.73 m^2^.

Elevated LDL-C was based on LDL-C concentration ≥3.0 mmol/L

Hypertension was based on a clinical diagnosis code, systolic blood pressure ≥140 mmHg and/or diastolic blood pressure ≥ 90 mmHg, and/or being on antihypertensive medication treatment.

*Definition of abbreviations*

CKD = chronic kidney disease; LDL-C = low-density lipoprotein cholesterol
